# Myristic acid selectively augments β‐tubulin levels in C2C12 myotubes via diacylglycerol kinase δ

**DOI:** 10.1002/2211-5463.13466

**Published:** 2022-07-27

**Authors:** Hiromichi Sakai, Ken‐ichi Matsumoto, Takeshi Urano, Fumio Sakane

**Affiliations:** ^1^ Department of Biosignaling and Radioisotope Experiment, Interdisciplinary Center for Science Research, Organization for Research and Academic Information Shimane University Izumo Japan; ^2^ Department of Biochemistry Shimane University School of Medicine Izumo Japan; ^3^ Department of Chemistry, Graduate School of Science Chiba University Japan

**Keywords:** diacylglycerol kinase, myotube, myristic acid, type II diabetes, β‐tubulin

## Abstract

Effective amelioration of type II diabetes requires therapies that increase both glucose uptake activity per cell and skeletal muscle mass. Myristic acid (14:0) increases diacylglycerol kinase (DGK) δ protein levels and enhances glucose uptake in myotubes in a DGKδ‐dependent manner. However, it is still unclear whether myristic acid treatment affects skeletal muscle mass. In this study, we found that myristic acid treatment increased the protein level of β‐tubulin, which constitutes microtubules and is closely related to muscle mass, in C2C12 myotubes but not in the proliferation stage in C2C12 myoblasts. However, lauric (12:0), palmitic (16:0) and oleic (18:1) acids failed to affect DGKδ and β‐tubulin protein levels in C2C12 myotubes. Moreover, knockdown of DGKδ by siRNA significantly inhibited the increased protein level of β‐tubulin in the presence of myristic acid, suggesting that the increase in β‐tubulin protein by myristic acid depends on DGKδ. These results indicate that myristic acid selectively affects β‐tubulin protein levels in C2C12 myotubes via DGKδ, suggesting that this fatty acid improves skeletal muscle mass in addition to increasing glucose uptake activity per cell.

## Graphical abstract

Myristic acid (14:0, MA) increases diacylglycerol kinase (DGK) δ protein levels and enhances glucose uptake in myotubes in a DGKδ‐dependent manner. In this study, we found that MA also augments β‐tubulin protein levels through DGKδ in C2C12 myotubes. Because β‐tubulin is closely related to muscle mass, MA may improve both glucose uptake per cell and skeletal muscle mass.

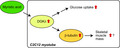

**Abbreviations**
DGK: diacylglycerol kinase
GAPDH: glyceraldehyde‐3‐phosphate dehydrogenase
MyHC: myosin heavy chain

The International Diabetes Federation reports that 536.6 million adults (20–79 years) have diabetes in 2021 worldwide, and the number is predicted to rise to 783.2 million by 2045 [1]. Type II diabetes is a metabolic disorder characterized by hyperglycemia, which is caused by insulin resistance. Skeletal muscle is the main organ of glucose uptake [2]. Therefore, in addition to decreased glucose uptake per cell, a reduction in skeletal muscle mass is well recognized as a factor that leads to insulin resistance, and in fact, sarcopenia, which is the loss of skeletal muscle mass caused by aging and/or immobility, is strongly associated with insulin resistance [3]. Therefore, insulin resistance and skeletal muscle mass loss are closely related. Consequently, to ameliorate type II diabetes, improvements in both glucose uptake per cell and skeletal muscle mass are important.

Diacylglycerol kinase (DGK) phosphorylates diacylglycerol and produces phosphatidic acid [4, 5, 6, 7]. Because diacylglycerol and phosphatidic acid are lipid second messengers, DGK regulates a wide variety of biological processes in mammalian cells by controlling the balance of these signaling lipids. Ten mammalian DGK isozymes have been identified, and these isozymes are classified into five groups (types I−V) based on their distinct structures [4, 5, 6, 7].

DGKδ, which belongs to the type II DGK family, including the δ, η and κ isoforms [8], is highly expressed in skeletal muscle [9] and myoblasts/myotubes [10]. Chibalin et al. [11] reported that a reduction in DGKδ expression decreased insulin‐mediated glucose uptake in skeletal muscle and consequently, exacerbated the severity of type II diabetes. Moreover, activation of DGKδ in response to high glucose levels, reduces protein kinase C‐α activity for glucose uptake in muscle cells [12]. Interestingly, DGKδ gene expression levels were increased in skeletal muscles after exercise training, which improves muscle mass and insulin sensitivity [13, 14]. These observations indicate that DGKδ plays a key role in glucose uptake in skeletal muscle.

Notably, we have recently reported that chronic administration of myristic acid (a 14‐carbon saturated fatty acid (14:0)), which is a saturated fatty acid, increases DGKδ protein levels *in vivo* in skeletal muscle of Nagoya‐Shibata‐Yasuda mice, which are known as a mouse model of type II diabetes, and reduces blood glucose levels during glucose tolerance tests and insulin‐responsive blood glucose levels [15]. We have already found that myristic acid increases DGKδ protein levels and augments glucose uptake in muscle cells in a DGKδ‐dependent manner [16, 17]. However, the effects of myristic acid on skeletal muscle mass are still unclear.

β‐Tubulin is closely related to skeletal muscle mass [18, 19, 20, 21]. Moreover, expression of the β‐tubulin gene is upregulated by exercise training [13]. Therefore, in the present study, we investigated whether myristic acid affects the protein levels of β‐tubulin in myotubes. We found that myristic acid selectively and substantially increased β‐tubulin levels in a DGKδ‐dependent manner.

## Materials and methods

### Cell culture

Mouse myoblasts (C2C12 cell line) were maintained on 100‐mm dishes in growth medium [Dulbecco's modified Eagle's medium (Wako Pure Chemicals, Osaka, Japan) containing 10% fetal bovine serum (Gibco, Carlsbad, CA, USA)] at 37 °C in an atmosphere containing 5% CO_2_. For differentiation into myotubes, 90% confluent C2C12 myoblasts grown on 60‐mm dishes were cultured in differentiation medium (Dulbecco's modified Eagle's medium containing 0.1% fetal bovine serum and 5 μg·mL^−1^ insulin; Sigma–Aldrich, St. Louis, MO, USA) for 3 days.

### RNA interference

To silence the expression of mouse DGKδ, Stealth RNAi duplexes (5′‐GAAUGUGAUGCUGGAUCUUACUAAA‐3′ and 5′‐UUUAGUAAGAUCCAGCAUCACAUUC‐3′; Invitrogen, Carlsbad, CA, USA) were used [22]. The duplex was transfected into C2C12 myoblasts by electroporation (at 350 V and 300 μF) using a Gene Pulser Xcell™ Electroporation System (Bio–Rad Laboratories, Hercules, CA, USA). The transfected cells were grown in growth medium for 48 h; then, the medium was replaced with differentiation medium.

### Fatty acid treatment

Lauric acid (12:0, Nacalai Tesque, Kyoto, Japan), myristic acid (Wako Pure Chemicals), palmitic acid (16:0, Wako Pure Chemicals) and oleic acid (18:1, Nacalai Tesque) were dissolved in DMSO. To generate bovine serum albumin‐bound fatty acids, each fatty acid was mixed with an equal volume of phosphate‐buffered saline containing 10% (w/v) fatty acid‐free bovine serum albumin (Wako Pure Chemicals). After sonication, the mixture was added to cell culture medium (0.5% v/v final concentration of DMSO), and the cells were further incubated for 24–48 h. The cultured cells were harvested for western blot analysis.

### Western blotting

The cells grown under each culture condition were washed by cold phosphate‐buffered saline and were directly harvested with 1× SDS sample buffer. The cells were sonicated by Branson Sonifier 450 (Branson Ultrasonics, Danbury, CT, USA). The homogenate (5–20 μL of each sample) was separated on SDS/PAGE gels for western blotting with anti‐DGKδ [9], anti‐cyclin D1 (sc‐450, Santa Cruz Biotechnology, Santa Cruz, CA, USA), anti‐cyclin D3 (sc‐182, Santa Cruz Biotechnology), anti‐myogenin (sc‐12732, Santa Cruz Biotechnology), anti‐myosin heavy chain (MyHC; MF20, Developmental Studies Hybridoma Bank, Iowa City, IA, USA), anti‐β‐tubulin (T4026, Sigma–Aldrich), anti‐β‐actin (A5441, Sigma–Aldrich) and anti‐glyceraldehyde‐3‐phosphate dehydrogenase (GAPDH; 016‐25523, Wako Pure Chemicals) antibodies. The immunoreactive bands were visualized using horseradish peroxidase‐conjugated anti‐rabbit or anti‐mouse IgG antibody (Cell Signaling Technology, Danvers, MA, USA) and Pierce™ ECL Western Blotting Substrate (Thermo Scientific, Waltham, MA, USA). The intensity of each band was measured using Amersham™ ImageQuant™ 800 (GE Healthcare, Chicago, IL, USA).

### Statistics

The data are presented as the mean ± SD. Statistical analysis was performed using a two‐tailed *t* test for the comparison of two groups or ANOVA followed by Dunnett's test or Student–Newman–Keuls test for multiple comparisons to determine any significant differences. *P* < 0.05 was considered significant.

## Results

### Myristic acid selectively increases β‐tubulin levels in C2C12 myotubes

**Fig. 1:**
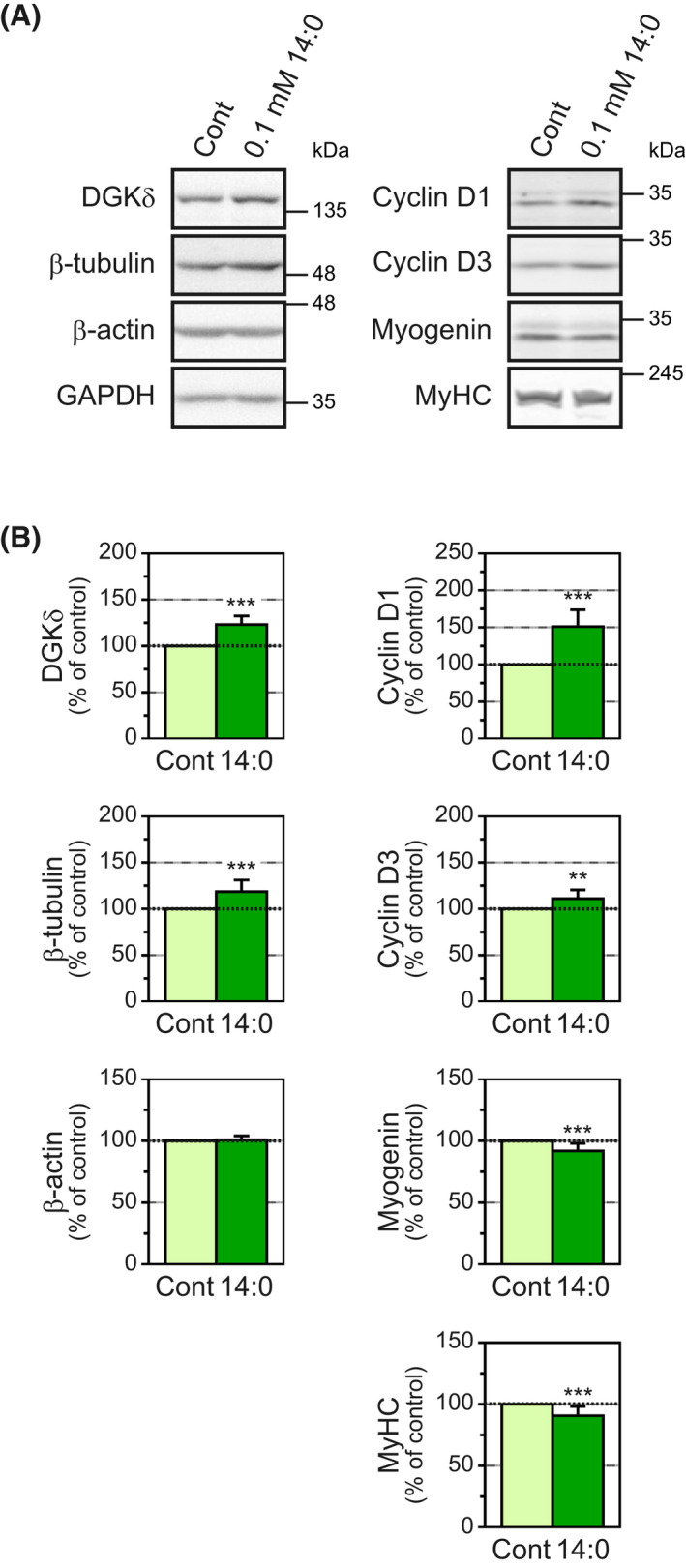
Effects of myristic acid on several protein levels in C2C12 myotubes. (A) C2C12 myotubes at 48 h of culture in differentiation medium were treated for 24 h with 0.1 mm myristic acid (14:0). DGKδ, β‐tubulin, β‐actin, GAPDH, cyclin D1, cyclin D3, myogenin and MyHC in the C2C12 cells treated with 0.1 mm myristic acid were detected by western blot. (B) The protein levels of each protein were normalized to the GAPDH level. The protein levels for the control were set as 100%. Values are presented as the means ± SD (*n* = 8). **P < 0.01 and ***P < 0.001 versus untreated cells, two‐tailed *t* test. Representative images (A) and graphs (B) of DGKδ, β‐tubulin, β‐actin and GAPDH are shown in left side for Section 3.1; Representative images (A) and graphs (B) of cyclin D1, cyclin D3, myogenin and MyHC are shown in right side for Section 3.2.

C2C12 myotubes at 48 h after myogenic differentiation were treated with 0.1 mm myristic acid in differentiation medium for 24 h. Because the levels of GAPDH protein/total proteins were not influenced by myristic acid, GAPDH was used as a control for western blot analysis. As previously reported [16, 17, 23], myristic acid treatment increased the levels of DGKδ protein (23.2% increase) compared with control treatment (Fig. 1A,B (left)). We next examined the amounts of β‐tubulin and β‐actin, which are cytoskeletal proteins that constitute microtubules and microfilaments, respectively. Notably, we found that myristic acid treatment significantly increased the protein levels of β‐tubulin (18.5% increase; Fig. 1A,B (left)). However, the fatty acid did not augment β‐actin levels (Fig. 1A,B (left)).

**Fig. 2:**
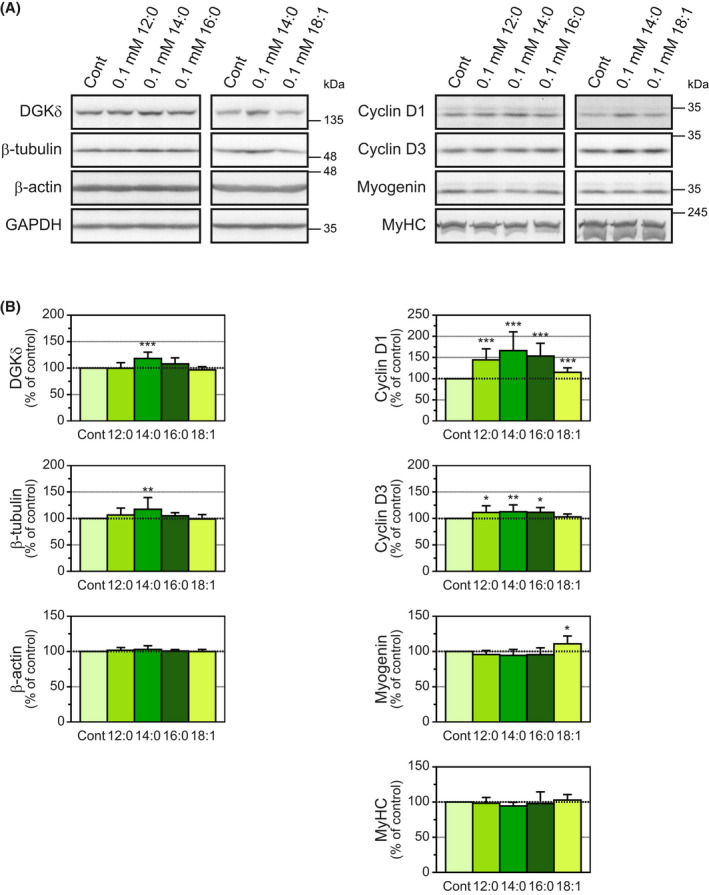
Effects of various fatty acids on the levels of several proteins in C2C12 myotubes. (A) C2C12 myotubes grown for 48 h in differentiation medium were treated for 24 h with 0.1 mm lauric (12:0), myristic (14:0), palmitic (16:0) or oleic (18:1) acid. DGKδ, β‐tubulin, β‐actin, GAPDH, cyclin D1, cyclin D3, myogenin and MyHC in the C2C12 cells treated with each fatty acid were detected by western blot. (B) The expression levels of each protein were normalized to the GAPDH level. The protein levels for the control were set as 100%. Values are presented as the means ± SD (*n* = 7–12). *P < 0.05, **P < 0.01 and ***P < 0.005 versus untreated cells, ANOVA followed by Dunnett's test. Representative images (A) and graphs (B) of DGKδ, β‐tubulin, β‐actin and GAPDH are shown in left side for Section 3.1; Representative images (A) and graphs (B) of cyclin D1, cyclin D3, myogenin and MyHC are shown in right side for Section 3.2.

To confirm whether the increase in β‐tubulin protein in C2C12 myotubes is myristic acid‐selective, C2C12 myotubes at 48 h after myogenic differentiation were treated with 0.1 mm lauric, palmitic and oleic acids for 24 h in addition to myristic acid. We verified that the increase in DGKδ protein (17.2% increase) was induced by myristic acid alone (Fig. 2A,B (left)), similar to our previous results [16, 17, 23]. Intriguingly, the protein levels of β‐tubulin were substantially increased in myotubes treated with myristic acid alone (18.3% increase) but not with lauric, palmitic or oleic acids (Fig. 2A,B (left)). The protein levels of β‐actin were not changed by any fatty acids (Fig. 2A,B (left)).

### Saturated fatty acids increase the amounts of cyclin D1 and D3 in C2C12 myotubes

DGKδ regulates C2C12 myogenic differentiation [22], and β‐tubulin is also associated with the formation and development of C2C12 myotubes [18, 19, 20]. Thus, we next examined the effects of myristic acid on the protein levels of differentiation markers. The levels of cyclin D1, which is a key enhancer of cell proliferation and is downregulated during myogenic differentiation [24], were increased by myristic acid treatment (51.0% increase). In addition, the protein levels of cyclin D3, which promotes exit from the cell cycle and induces differentiation [25] in contrast to cyclin D1, were also increased (10.9% increase; Fig. 1A,B (right)). The amounts of myogenin and MyHC, which are early‐ and late‐stage differentiation markers, respectively [26, 27, 28], were only slightly suppressed (8.3% decrease and 9.3% decrease, respectively; Fig. 1A,B (right)). Thus, it is likely that the effects of myristic acid on cyclin D3 and cyclin D1, which have opposite effects on each other, canceled each other out.

In addition to myristic acid, cell treatment with lauric and palmitic acids increased the protein levels of both cyclin D1 and cyclin D3 (lauric acid: 44.3% increase and 11.2% increase, respectively; myristic acid: 66.3% increase and 12.6% increase, respectively; and palmitic acid: 53.6% increase and 11.5% increase, respectively; Fig. 2A,B (right)). However, these saturated fatty acids only slightly reduced or did not substantially affect myogenin and MyHC protein levels (Fig. 2A,B (right)). Because saturated fatty acids (lauric, myristic and palmitic acids) commonly augment cyclin D1 and cyclin D3 levels, these effects are not myristic acid‐selective. Conversely, in oleic acid‐treated myotubes, myogenin levels were increased compared with control cells (10.8% increase; Fig. 2A,B (right)).

### Myristic acid does not increase β‐tubulin levels at the proliferation or early differentiation stages of C2C12 myoblasts

**Fig. 3:**
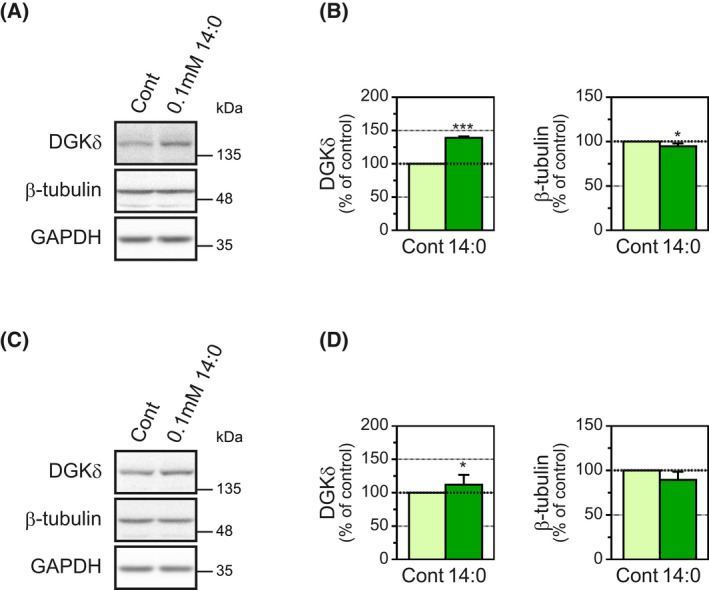
Effects of myristic acid on several protein levels in C2C12 cells during proliferation and at the early differentiation stage. (A) C2C12 myoblasts grown for 24 h in growth medium were treated for 24 h with 0.1 mm myristic acid. DGKδ, β‐tubulin, β‐actin and GAPDH in the C2C12 cells treated with 0.1 mm myristic acid were detected by western blot. (B) The expression levels of each protein were normalized to the GAPDH level. The protein levels for the control were set as 100%. Values are presented as the means ± SD (*n* = 4). *P < 0.05 and ***P < 0.005 versus untreated cells, two‐tailed *t* test. (C) After initiation of differentiation (at 0 h of differentiation), C2C12 cells were treated for 48 h with 0.1 mm myristic acid. DGKδ, β‐tubulin, β‐actin and GAPDH in the C2C12 cells treated with 0.1 mm myristic acid were detected by western blot. (D) The expression levels of each protein were normalized to the GAPDH level. The protein levels for the control were set as 100%. Values are presented as the means ± SD (*n* = 8). *P < 0.05 and ***P < 0.005 versus untreated cells, two‐tailed *t* test.

We next examined the effects of myristic acid treatment on β‐tubulin levels at the proliferation and early differentiation stages of C2C12 myoblasts. Myoblast cells grown for 24 h in growth medium were treated with 0.1 mm myristic acid in growth medium for 24 h. In myristic acid‐treated cells, the protein levels of DGKδ were increased (39.1% increase; Fig. 3A,B). However, the protein levels of β‐tubulin were not substantially increased (slightly decreased (5.3% decrease)) compared with those in control cells (Fig. 3A,B). Essentially, the same results were obtained at the early stage of differentiation (0–48 h after myogenic differentiation; Fig. 3C,D). These results indicate that the effect of myristic acid on β‐tubulin levels is selective in differentiated C2C12 cells (myotubes).

### Myristic acid augments β‐tubulin levels via DGKδ in C2C12 myotubes

**Fig. 4:**
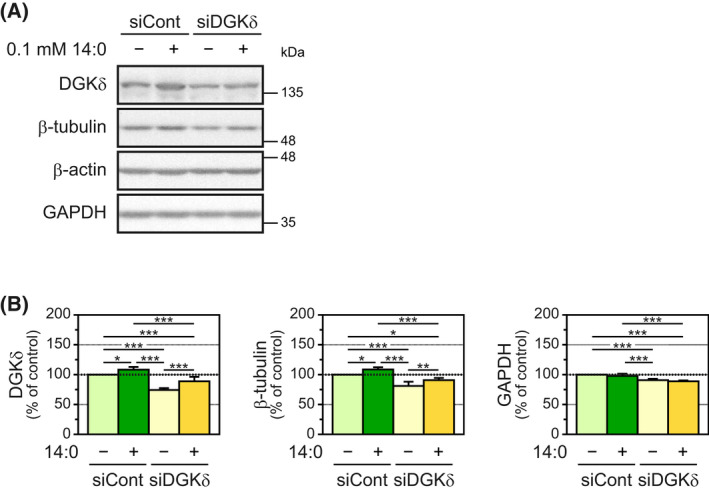
Effects of DGKδ silencing on β‐tubulin protein levels in C2C12 myotubes in the presence or absence of myristic acid. (A) C2C12 myoblasts transfected with control siRNA or DGKδ‐specific siRNA were grown for 48 h in growth medium; then, the medium was replaced with differentiation medium. C2C12 myotubes at 48 h after differentiation were treated for 24 h with 0.1 mm myristic acid, and DGKδ, β‐tubulin, β‐actin and GAPDH were detected by western blot. (B) The expression levels of each protein were normalized to the β‐actin level. The protein levels for the control (myristic acid, control siRNA) were set as 100%. Values are presented as the means ± SD (*n* = 4). *P < 0.05, **P < 0.01 and ***P < 0.005, ANOVA followed by Student–Newman–Keuls test.

Myristic acid treatment increased the protein levels of both DGKδ and β‐tubulin (Figs 1 and 2). Thus, we investigated whether the fatty acid regulated β‐tubulin protein levels in a DGKδ‐dependent manner. DGKδ‐siRNA affected GAPDH expression levels (Fig. 4A,B). Therefore, β‐actin was used as a control for western blot analysis because its levels were not changed by DGKδ‐siRNA (Fig. 4A). The protein levels of DGKδ in myotubes transfected with DGKδ‐siRNA were confirmed to be decreased (25.7% decrease) compared with those in myotubes transfected with control siRNA (Fig. 4A,B). β‐Tubulin protein levels were also reduced (19.9% decrease) by suppression of DGKδ expression. In addition, myristic acid treatment to cells transfected with control siRNA increased the protein levels of DGKδ and β‐tubulin (8.4% increase and 8.6% increase, respectively), and these levels were significantly attenuated by DGKδ‐siRNA (19.1% decrease and 17.3% decrease, respectively; Fig. 4A and B). These results strongly suggest that the upregulation of β‐tubulin protein levels by myristic acid is DGKδ‐dependent.

## Discussion

In this study, we found that myristic acid selectively increased β‐tubulin protein levels in C2C12 myotubes (Fig. 1). This effect has three characteristics: (a) fatty acid selectivity (Fig. 2), (b) differentiated cell (myotube)‐selectivity (Fig. 3) and (c) DGKδ dependency (Fig. 4).

Only myristic acid, but not lauric, palmitic or oleic acids, increased β‐tubulin protein levels (Fig. 2). This selectivity of myristic acid is similar to its effect on DGKδ protein levels in C2C12 myotubes [16, 17, 23]. In contrast to the effects of myristic acid on β‐tubulin and DGKδ protein levels, all saturated fatty acids examined affected the protein levels of cyclin D1 and cyclin D3 and caused a slight inhibition of differentiation (Figs 1 and 2), indicating that the differentiation‐related changes are not myristic acid‐selective. Lee et al. [29] showed that oleic acid treatment accelerates myogenic differentiation. In this study, we also observed increased expression levels of myogenin (Fig. 2). However, oleic acid treatment did not affect the protein levels of DGKδ or β‐tubulin, suggesting that the effect of the fatty acid is independent of DGKδ and β‐tubulin.

Increases in β‐tubulin protein levels induced by myristic acid were not observed at the proliferation or early differentiation stages of myoblasts (Fig. 3). The results suggest that this event is selective for differentiated cells (myotubes). In contrast to β‐tubulin, DGKδ was also increased by myristic acid in proliferating cells (Fig. 3). Thus, the link between DGKδ and β‐tubulin protein levels may be lost in proliferating C2C12 cells. It is interesting to reveal the mechanisms conferring the difference between proliferating and differentiated C2C12 cells.

As described above, myristic acid, but not lauric, palmitic or oleic acid, increased the amount of β‐tubulin, similar to the DGKδ protein [16, 17, 23]. Moreover, previous studies have shown that exercise training increased both DGKδ [13, 14] and β‐tubulin [13] protein levels in skeletal muscles. These results imply a physiological correlation between β‐tubulin and DGKδ. Indeed, an increase in β‐tubulin protein by myristic acid treatment likely occurred in a DGKδ‐dependent manner (Fig. 4). Because β‐tubulin (microtubule) density is almost the same in muscle fibers [21], the upregulation of β‐tubulin levels could be correlated with the increase in skeletal muscle mass. In addition, microtubules, including β‐tubulin, are involved in muscle cell elongation and fusion [18, 19, 20], which also contribute to skeletal muscle mass increases. Therefore, it is possible that the increase in β‐tubulin due to increased expression of DGKδ as a result of myristic acid treatment promotes skeletal muscle mass increases. Unfortunately, it remains unclear how myristic acid selectively regulates DGKδ/β‐tubulin levels, and further study is needed to clarify this mechanism. Moreover, in the present study, we showed that myristic acid increased β‐tubulin protein levels. However, a direct relationship between increased β‐tubulin protein levels and skeletal muscle mass is still unclear. Future studies using *in vivo* experiments may clarify this relationship.

Protein synthesis and degradation are known to be important for skeletal muscle formation and maintenance. However, not only protein synthesis/degradation but also various factors are associated with muscle formation and maintenance. We previously demonstrated that DGKδ is not associated with the Akt/mTOR signal pathway [22], which is related to protein synthesis [30, 31]. Thus, in the present study, we aimed to discover which factors for muscle development are regulated by myristic acid in a DGKδ‐dependent manner and found the increase in β‐tubulin protein levels, which, in general, is not directly associated with protein synthesis/degradation.

Our previous study showed that downregulation of DGKδ protein levels increases cyclin D1 expression [22], indicating that suppression of DGKδ inhibits myogenic differentiation. However, in this study, myristic acid increased the expression levels of cyclin D1, which accelerates cell proliferation [24], and cyclin D3, which, conversely, promotes cell differentiation [25]; myristic acid also upregulated DGKδ protein levels (Figs 1 and 2). The amounts of cyclin D1/D3 proteins were controlled by not only myristic acid but also lauric and palmitic acids (Fig. 2), indicating that this event is not myristic acid‐selective. Therefore, these results suggest that saturated fatty acids, including myristic acid, regulate cyclin D1/D3 protein levels in a DGKδ‐independent manner.

Saturated fatty acids have been reported to have substantial negative effects, such as apoptotic and antiproliferative effects, on various cultured cells [32]. For example, palmitic acid suppresses the proliferation of C2C12 myoblasts and inhibits their differentiation [33]. We observed that myristic acid also apparently inhibits the proliferation and early‐stage differentiation of C2C12 myoblasts (Sakai et al. unpublished work), suggesting that these negative effects in C2C12 cells may be common among saturated fatty acids. On the other hand, we showed that the inhibition of C2C12 myogenic differentiation at late‐stage differentiation by myristic acid was very weak (Figs 1 and 2). These results indicate that the effects of myristic acid are different for C2C12 myoblasts in late‐stage differentiation, early‐stage differentiation and proliferation. Because skeletal muscles in adults have already differentiated, it seems that the negative effects of myristic acid are not a considerable issue in adults. In addition, palmitic acid is far more abundant than myristic acid in human plasma [34], and consequently, the negative effects of saturated fatty acids are expected to be constantly saturated by palmitic acid. In general, the negative effects of saturated fatty acids are known to be canceled and balanced by the positive effects of unsaturated fatty acids, such as oleic and linoleic (18:2) acids [29], which are abundant in the plasma, as well as palmitic acid [34]. For the abovementioned reasons, it is likely that chronic administration of myristic acid does not have considerable negative effects on skeletal muscle cells in adults. Importantly, the upregulation of β‐tubulin protein levels through DGKδ is myristic acid‐selective, and therefore, it is possible that myristic acid specifically causes an effective increase in muscle mass.

In summary, we found that myristic acid selectively increases β‐tubulin protein levels in a DGKδ‐dependent manner. β‐Tubulin is associated with skeletal muscle mass [18, 19, 20, 21]. Therefore, myristic acid can be a useful nutritional supplement that improves hyperglycemia not only by enhancing glucose uptake per cell [15, 16, 17] but also by increasing skeletal muscle mass, which can also prevent sarcopenia. However, to reveal the effects of myristic acid in more detail, it is important to further investigate skeletal muscle features, including muscle mass, in mice administered myristic acid.

## Conflict of interest

The authors declare no conflict of interest.

## Author contributions

HS designed and performed the experiments and analyzed the data. HS and FS designed the study, supervised all aspects of data collection and wrote the manuscript. TU and KM provided advice on the experimental design and critically revised the manuscript for intellectual content. All authors read and approved the final manuscript.

## Data Availability

The data that support the findings of this study are available from the corresponding authors upon reasonable request.
